# Refractory Hypothyroidism: Unexpected Outcome During Treatment of Giant Cell Arteritis

**DOI:** 10.1002/ccr3.71289

**Published:** 2025-10-21

**Authors:** Elise Deprince, Solange Grunenwald, Celine Mouly, Julie Benoit, Philippe J. Caron

**Affiliations:** ^1^ Department of Endocrinology, Metabolic Diseases and Nutrition, Cardiovascular and Metabolic Unit Larrey University Hospital Toulouse France

**Keywords:** gastrointestinal absorption, giant cell arteritis, *Helicobacter pylori*, hypothyroidism, intestinal gastritis, refractory hypothyroidism

## Abstract

Refractory hypothyroidism is not uncommon in patients treated with oral levothyroxine, and it represents a challenge for clinicians as regards a physiopathological diagnosis and the most appropriate long‐term therapeutic management in order to restore normal TSH concentrations.

## Graphical abstract

Refractory hypothyroidism in a patient with giant cell arteritis: doses of levothyroxine and serum TSH levels during long term follow‐up.

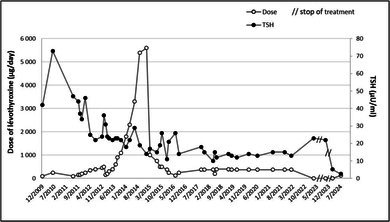

## 1 Introduction

Hypothyroidism is a common endocrine disease. The usual treatment is levothyroxine (LT4) administered orally, with the dose being dependent on the severity of the thyroid disease (on the decrease of the patient's concentration of thyroxine or T4) and on the patient's weight. The average dosage for postoperative hypothyroidism is between 1.6 and 1.8 g/kg/day to restore normal thyroid function.

However, in some patients, treating hypothyroidism can be difficult. In such patients, it is necessary to rule out any possible causes of the need for a high dose of levothyroxine, including drug or food interactions, cirrhosis, weight gain, nephrotic syndrome, and pregnancy [1, 2]. Refractory hypothyroidism, which affects 15%–20% of patients treated with levothyroxine [3], is defined as TSH levels persisting beyond the upper limit of the normal range (in primary hypothyroidism caused by damage to the thyroid gland) despite levothyroxine doses higher than 1.9 μg/kg/day [1]. The most common causes of refractory hypothyroidism are failure to comply with treatment, gastrointestinal conditions leading to malabsorption of levothyroxine, and increased clearance of exogenous levothyroxine.

A levothyroxine absorption test is the “gold standard” for differentiating between impaired gastrointestinal absorption of levothyroxine and poor compliance with treatment or increased clearance of exogenous levothyroxine [4, 5, 6]. Nevertheless, the cause of refractory hypothyroidism cannot be identified in 10%–20% of such patients [3].

We describe here the case of a female patient with postoperative refractory hypothyroidism in the context of an autoimmune disease, whose levothyroxine dosage requirements and absorption rate became normalized during the treatment of inflammatory giant cell arteritis. We discuss several hypotheses relating to the pathophysiology of the refractory hypothyroidism in the patient.

## 2 Case History/Examination

A 65‐year‐old female patient is followed up for the treatment of postoperative hypothyroidism. Her medical history includes a total thyroidectomy in November 2008 in the context of autoimmune thyroid disease (positive TPO antibodies) with alternating episodes of hypothyroidism and thyrotoxicosis as well as orbitopathy, inflammatory rheumatism treated since 2014 with hydroxychloroquine sulfate (400 mg/day), then methotrexate (10–15 mg/week) and corticosteroid therapy at a dose of 1 mg/kg/day during flare‐ups, and lastly osteoporosis with fractures treated with zoledronic acid (five infusions of 5 mg between 2014 and 2021).

**FIGURE 1:**
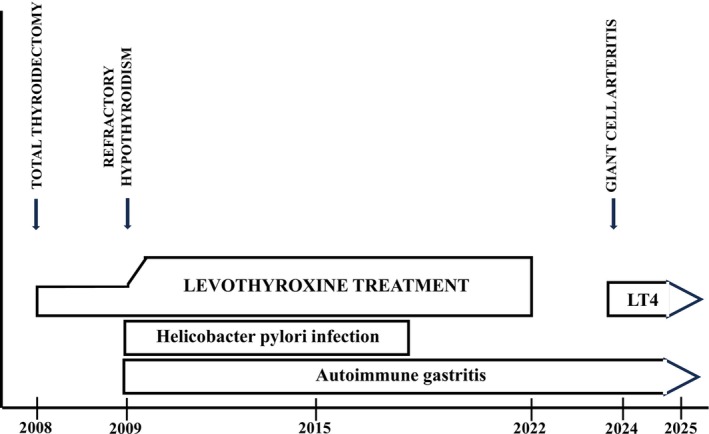
Timeline of medical history of the patient with refractory hypothyroidism before and during giant cell arteritis treatment.

After the total thyroidectomy, the patient was started on replacement therapy with levothyroxine (Merck Serono Laboratory; 100 μg/day for a body weight of 73 kg, i.e., 1.3–1.4 μg/kg/day), which initially resulted in normal postoperative thyroid function (shown in Figure 1). In December 2009, a TSH concentration of 42 μIU/mL led to a gradual increase in her levothyroxine dose, reaching 250 g/day (3.4 μg/kg/day). Despite increasing the levothyroxine dose, the patient's TSH levels did not normalize.

**FIGURE 2:**
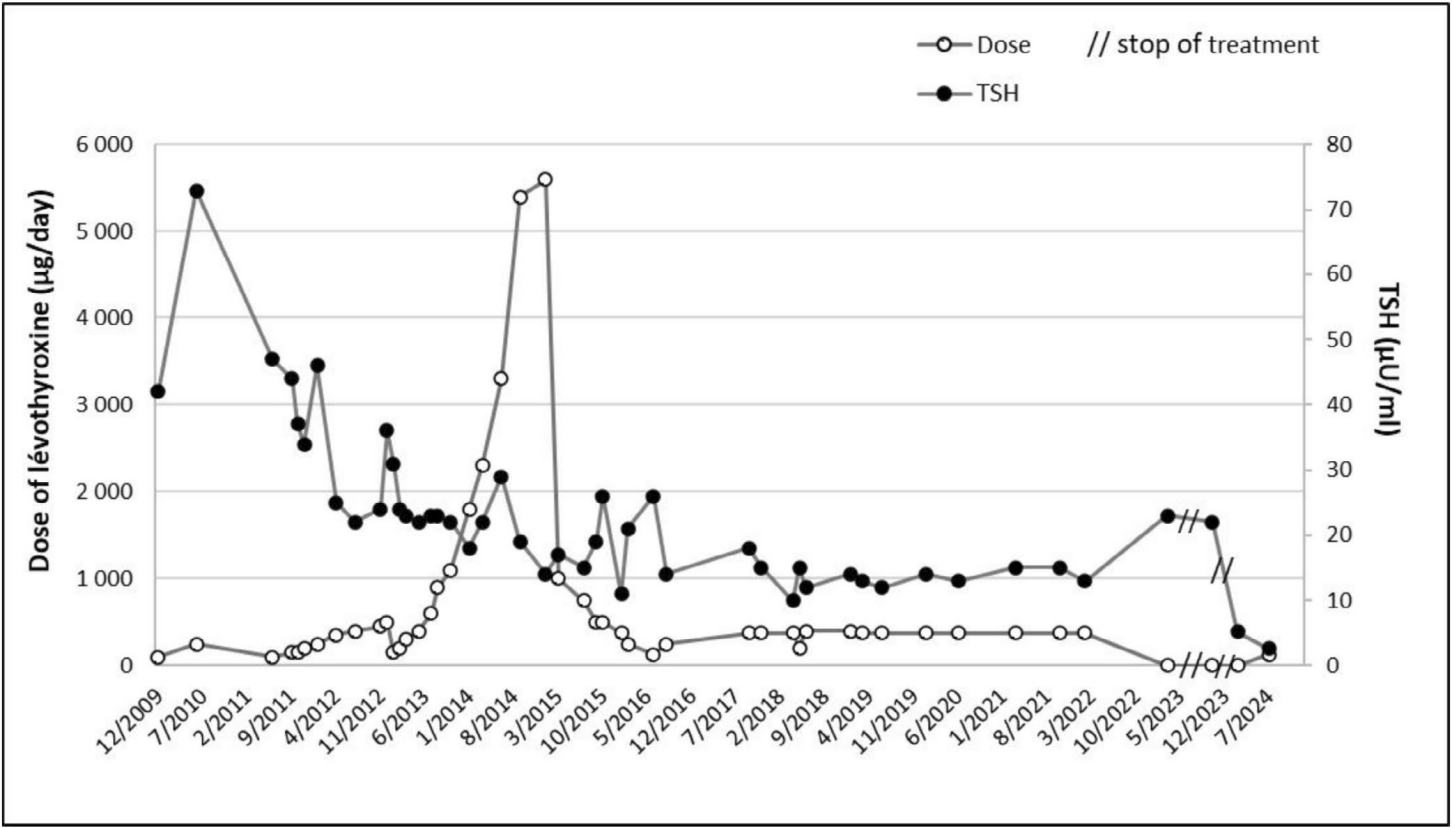
Doses of levothyroxine and serum TSH concentrations during long‐term follow‐up of the patient with refractory hypothyroidism, before and during giant cell arteritis.

An assessment of etiology showed a *Helicobacter pylori* infection as well as autoimmune gastritis, with gastric parietal cell antibodies. Changing the formulations of the levothyroxine (tablets, liquid solution, soft capsules) and increasing the dose to 5600 μg/day in 2015 (120 drops of levothyroxine + 5000 μg in tablets) had no effect on the symptoms described by the patient (asthenia, hair loss, hair loss at the end of the eyebrows, sensitivity to cold, weight gain, moon face, and alternating diarrhea/constipation) and did not restore normal TSH levels (shown in Figure 2).

Follow‐up assessments revealed a chronic *Helicobacter pylori* infection between 2011 and 2015, with several failed eradication attempts using antibiotics. However, urea breath tests and gastroscopy performed after 2015 showed that the *Helicobacter pylori* infection was eradicated successfully (shown in Figure 1).

In addition, an interference in the TSH level could be seen following precipitation by polyethylene glycol (PEG), indicating a macro‐TSH. The patient stopped taking levothyroxine therapy in December 2022, while TSH concentrations were in the range of 12–15 mU/L with a high dose (375 μg/day) of levothyroxine treatment (shown in Figures 1 and 2). After levothyroxine withdrawal, the TSH levels slightly increased whereas free T4 concentrations moderately decreased (Table 1). Central hypothyroidism was ruled out based on a normal hormonal pituitary profile and an MRI scan of the pituitary sella.

**TABLE 1:** Thyroid parameters during the follow‐up of the woman with refractory hypothyroidism. Normal range of thyroglobulin (Tg) concentration: 3.5–25 ng/mL, and of anti‐Tg antibodies (< 115 IU/mL).

Date	06/2020	03/2021	07/2022	03/2023	10/2023	07/2024	01/2025
Levothyroxine treatment (μg/day)	375	375	375	0	0	125	125
TSH (mU/L)	13	15	13	23	22	2.6	0.019
Free T4 (pg/mL)	7.1	6.2	6.8	5.0	5.2	11.8	24.4*
Free T3 (pg/mL)	2.9	2.8	2.9	2.2	2.8	3.0	5.5*
Thyroglobulin (ng/mL)					0.65		
Anti‐Tg antibodies (IU/mL)					760		

* pmol/L.

The woman presented liquid diarrhea in December 2023 and a deterioration of her general condition with a 7‐kg weight loss during January 2024. A physical examination revealed induration of the temporal arteries, together with severe inflammation (CRP = 150 mg/L). An assessment revealed large‐vessel vasculitis in the form of clear hypermetabolism of the entire thoracic aorta, the subclavian arteries, the right brachiocephalic trunk, the primitive iliac arteries, and a part of the common carotid arteries, seen on an 18‐FDG PET scan, which led to the diagnosis of giant cell arteritis. In February 2024, corticosteroid therapy was started at 1 mg/kg/day (i.e., 70 mg/day), rapidly relieving the patient's diarrhea, and TSH concentration was 0.8 mU/L, free T4 free T4 = 11.9 pg/mL, and free T3 = 1.7 pg/mL, five days after the initiation of the corticosteroid therapy.

Three months later, a follow‐up 18‐FDG PET scan showed that the giant cell arteritis was still active, with persistent inflammatory aortitis, so corticosteroid therapy was stopped and treatment with a monoclonal antibody against interleukin‐6 (tocilizumab, 162 mg/week) was started in June 2024.

As the patient followed a levothyroxine treatment at 1.76 μg/kg/day, her thyroid function became normal from a clinical point of view, with normal levels of free T4 and TSH (shown in Figure 2).

Careful monitoring of the patient's thyroid function was instituted to adapt substitutive levothyroxine therapy: In January 2025, the control of thyroid parameters showed exogenous thyrotoxicosis (Table 1), and the levothyroxine dose was reduced (112 μg/day).

## 3 Differential Diagnosis, Investigation, and Treatment

Any intake conditions, compliance with treatment, drug and food interactions, and other factors that could lead to an increase in levothyroxine needs were examined and ruled out.

A levothyroxine absorption test performed in November 2011 showed a decreased absorption rate [39.5% (normal > 60%) after 200 μg of levothyroxine administered while fasting] [5].

**FIGURE 3:**
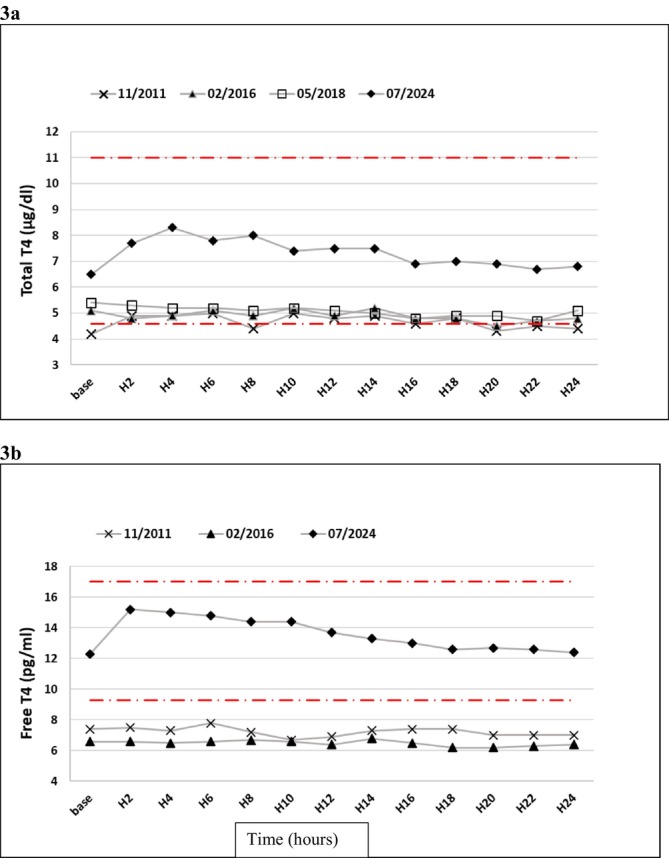
Total (Figure 3a) and free (Figure 3b) T4 concentrations during levothyroxine absorption tests of the patient with refractory hypothyroidism, before and during giant cell arteritis treatment. The *x*‐axis is the time (hours) after oral absorption of the levothyroxine dose.

Another levothyroxine absorption test showed a severely impaired absorption rate (7.9% after taking 500 μg of levothyroxine in the morning). Levothyroxine absorption tests performed in 2016 and 2018 showed a persistent levothyroxine absorption impairment (shown in Figure 3).

In the light of clinical symptoms of hypothyroidism and a normal levothyroxine absorption test (117% after a dose of 1000 μg), treatment with levothyroxine was reintroduced at a dosage that was increased to 125 μg/day.

In July 2024, a levothyroxine absorption test with 125 μg of levothyroxine confirmed a normal absorption rate (shown in Figure 3).

## 4 Conclusion and Results

In summary, refractory hypothyroidism is not a rare clinical problem in patients treated with levothyroxine, and it represents for clinicians a challenge of physiopathological diagnosis and long‐term therapeutic management. We describe here the case of a female patient with postoperative refractory hypothyroidism against the background of an autoimmune disease and whose levothyroxine dosage requirements became normalized, with restoration of euthyroidism during medical treatment of giant cell arteritis.

## 5 Discussion

Although most patients with hypothyroidism who undergo oral treatment with levothyroxine achieve normal thyroid function, refractory hypothyroidism—while it is far from an exception in clinical practice—requires performing an etiologic investigation and adapting the treatment. Nevertheless, the cause of refractory hypothyroidism cannot be identified in 10%–20% of such patients [3].

Normal gastric secretion is necessary for levothyroxine to be absorbed [7]. Our patient's decreased gastrointestinal absorption of levothyroxine can at least partly be explained by her chronic *Helicobacter pylori* infection between 2011 and 2015 and autoimmune gastritis diagnosed in 2011, which caused low stomach pH. Centanni et al. have reported an additive effect of a *H. pylori* infection and autoimmune gastritis on decreased stomach acid and levothyroxine absorption disorders [8].

Despite eradicating the *Helicobacter pylori* infection, levothyroxine absorption tests performed in 2016 and 2018 showed a persistent levothyroxine absorption impairment in the patient with autoimmune gastritis.

Depending on the severity of autoimmune gastritis, levothyroxine absorption can vary in such patients. Gonzales et al. [9] described the case of a male patient with autoimmune atrophic gastritis and a levothyroxine absorption rate of 0%, while Subramaniam et al. [10] discussed the case of a male patient with autoimmune atrophic gastritis and a levothyroxine absorption rate of 36.5% and two patients with a *H. pylori* infection with absorption rates of 30.6% and 50.5%, respectively.

Apart from decreased gastric secretion against a background of an autoimmune disease, another hypothesis as to the pathophysiology of the condition could be impaired function or expression of levothyroxine membrane transporters or the presence of cell membrane transporter antibodies. After levothyroxine withdrawal, the TSH levels slightly increased whereas free T4 concentrations moderately decreased. In the context of the patient with macro‐TSH, significant titers of anti‐thyroglobulin antibodies, and autoimmune disorders, one hypothesis is that slightly and moderately changes in TSH and free T4 levels after levothyroxine therapy withdrawal can be in relation to interferences in immunoassays of thyroid function parameters as previously reported [11, 12]. On the other hand, thyroglobulin concentration was low (0.65 ng/mL) after 10 month levothyroxine withdrawal, in favor of the absence of residual thyroid tissue after total thyroidectomy, and hormonal pituitary profile and MRI scan of the pituitary sella were normal, ruling out central hypothyroidism or pituitary disorders.

Few articles in the literature have raised the hypothesis of a levothyroxine absorption disorder linked to impaired function or expression of levothyroxine membrane transporters. In 2021, Chung et al. [13] published a female patient with levothyroxine malabsorption (8.4% absorption rate), with decreased expression of thyroid hormone membrane transporters in the distal ileum compared to a control group without any thyroid disorders. The 8.4% absorption rate found in this patient is comparable to our patient's absorption rate in 2016 (5.5%). Studies must be conducted to confirm the role of impaired expression or function of membrane transporters of the intestinal mucosa as a possible cause of levothyroxine malabsorption in patients with refractory hypothyroidism and autoimmune disease.

The role of abnormal gut microbiota is another possibility worth exploring. Yao et al. have reported that gut microbiota could affect the permeability and integrity of the intestinal barrier and that the doses of levothyroxine required to maintain stable TSH levels in patients with hypothyroidism could be linked to the patients having different gut microbiota [14].

Lastly, the hypothesis for why the patient's absorption rate normalized in February and July 2024 is the presence of inflammatory bowel disease due to giant cell arteritis (with the patient's sensitivity to corticosteroid therapy) [15, 16, 17], which caused the patient's digestive wall to become more permeable and the restoration of levothyroxine absorption. Moreover, during chronic treatment with a monoclonal antibody against interleukin 6 of inflammatory giant cell arteritis, the levothyroxine doses required by the patient were monitored and recently decreased, so that oral treatment with levothyroxine was adapted to maintain normal thyroid function.

## Author Contributions

**Elise Deprince:** data curation, writing – original draft. **Solange Grunenwald:** validation, writing – review and editing. **Celine Mouly:** writing – review and editing. **Julie Benoit:** writing – review and editing. **Philippe J. Caron:** conceptualization, validation, writing – review and editing.

## Consent

The authors confirm that written informed consent was obtained from the patient for publication of all data in the scientific manuscript.

## Conflicts of Interest

The authors declare no conflicts of interest.

## Data Availability

The data that support the findings of this study are available from the corresponding author upon reasonable request.
